# A New Species of the Feather Mite Genus *Grallolichus* Gaud, 1960 (Acariformes: Pterolichidae): First Report of a Commensal Mite Specific to the Sungrebe (*Heliornis fulica*)

**DOI:** 10.3390/ani14203035

**Published:** 2024-10-19

**Authors:** Jacek Dabert

**Affiliations:** Department of Animal Morphology, Faculty of Biology, Adam Mickiewicz University, Uniwersytetu Poznańskiego 6, 61-614 Poznań, Poland; dabert@amu.edu.pl

**Keywords:** feather mites, commensals, new taxa, morphology, host specificity, *Grallolichus*, ornithological collections

## Abstract

**Simple Summary:**

Feather mites are a specific group of ectoparasites or commensals of almost all bird species worldwide, morphologically and biologically adapted to function in a very unusual environment—the plumage of their hosts. They are able to attach themselves so strongly to feathers that they do not leave their hosts even after their death, remaining on or in the feathers as dried ‘mummies’. This makes it possible to discover and describe them by viewing birds preserved in museum collections. Of the currently known approximately 2500 species of feather mites, the vast majority are derived from just such a source of material. Also, the species described in this paper, new to knowledge, *Grallolichus heliornisi* sp. n. (Pterolichidae), comes from an old exhibition specimen of the sungrebe (*Heliornis fulica*) from the family finfoots (Heliornithidae), found in the collection of the Zoological Museum in Kiel (Germany). This is the first record of a representative of this genus on finfoots and the first description of a new species for *Grallolichus* in more than 50 years. The diagnosis of the new species is based on a detailed analysis of the morphology, complemented by a key to the species known so far, and summarized through a reflection on the importance of old ornithological collections for understanding the biodiversity and evolution of feather mites.

**Abstract:**

Feather mites of finfoots (Heliornithidae), a small gruiform family, are poorly and partly erroneously recognized. *Grallolichus heliornisi* sp. n. (Astigmata: Pterolichidae) is here described from the sungrebe *Heliornis fulica* as the first representative of the genus commonly found on close relatives of finfoots, Rallidae and Sarothuridae. This species belongs to the species group having ornamented dorsal shields and is morphologically most close to *G. proctogamus* inhabiting Eurasian coot (*Fulica atra*). Males of the new species differ from *G. proctogamus* mainly by the shape of opisthosomal lobes (triangular vs. rounded) and the aedeagus form (parallel sided vs. tapering distally). Females differ mainly by the shape of supranal concavity (open anteriorly vs. closed) and location of setae *h1* in relation to supranal concavity (lateral vs. anterior). A key to known species of the genus *Grallolichus* is provided. The morphological analysis and descriptive characterization of this species, like much of the approximately 2500 feather mite species described to date, were based on mummified mite material preserved in 19th-century old museum bird specimens. These often-forgotten collections are the only source for the analysis of the acarofauna of many rare, unavailable wild or even extinct bird taxa.

## 1. Introduction

Feather mites (Acariformes: Astigmata) are obligate commensals or parasites of all recent bird orders, where they inhabit various parts of the plumage but also skin and respiratory tracts of hosts 411]. Feather mites show an astonishing variety of morphological adaptations to the specific physical conditions prevailing in different locations on the birds’ bodies, resulting in the convergent origin of several morphotypes found in these microhabitats: down mites, vane mites, quill mites, skin mites, and nasal mites [1,2]. Sometimes, additional morphotypes are distinguished from quill walls, subcutaneous tissues, and nests (secondary nidicolous mites) [3].

Feather mites have traditionally been classified into two superfamilies, Analgoidea and Pterolichoidea, within the Psoroptidia clade, which groups almost exclusively commensals or parasites of mammals and birds [4,5]. Molecular phylogenies of the Acariformes, where feather mites have been relatively well sampled, suggest that this is an ecological group rather than a natural taxon [6,7,8]. Pterolichoidea are the most basal group of psoroptidian mites of which diverge the Analgoidea. In turn, the Sarcoptoidea, parasites of mammals, are nested within Analgoidea. Some studies suggest that the Pterolichoidea are monophyletic and the Analgoidea are paraphyletic [9]. The beginning of feather mite diversification dates to 145 MYA and is correlated with the appearance of the first flying bird-like dinosaurs [7].

Finfoots (Heliornithidae) are a family of gruiform birds, most closely related to flufftails (Sarothuridae), with which they form a sister clade to rails (Rallidae) [10]; the clade of these three families is sometimes considered a superfamily, Ralloidea [11]. These birds are found only in the tropical forests of Africa, Asia, and South and Central America, where they inhabit a variety of water-related habitats such as swamps, mangroves, and slow-moving or large bodies of water. This small family includes only three species within three monotypic genera: African finfoot (*Podica senegalensis*), masked finfoot (*Heliopais personatus*), and sungrebe (*Heliornis fulica*). Various aspects of the biology and ecology of these secretive birds are poorly understood, including the composition of their acarofauna.

The mites inhabiting the plumage of *Podica senegalensis* are best recognized. Three species are known from this bird: *Grallobia gauduini* Gaud et Mouchet, 1963 (Pterolichidae), *Gymnalloptes pallens* (Trouessart et Neumann, 1888) (Xolalgidae), and *Homeobrephosceles discosurus* (Trouessart, 1886) (Alloptidae). The feather mites of the other two finfoot species are poorly identified. No mites have so far been found on *Heliopais personata*, and one species, *Trouessartia fulicae* Berla, 1962 (Trouessartiidae), has been described from *Heliornis fulica*. However, according to Santana [12], this description was made based on three individuals belonging to three different *Trouessartia* species of unknown real hosts, probably because of accidental contamination, and, therefore, *T. fulicae* should be treated as *species inquirenda*. In fact, about 170 known species of the family Trouessartiidae populate mainly birds of the orders Passeriformes, with only a few scattered examples of successful colonization in other bird groups, such as Piciformes, Coraciiformes, and Caprimulgiformes [1]. Thus, this record is probably the result of an accidental contamination.

So far, 15 species of the genus *Grallolichus* Gaud, 1960 have been described (Table 1). Mites of this genus, along with its sister genus, *Grallobia* Hull, 1934, are the only representatives of the superfamily Pterolichoidea inhabiting the plumage of rails and flufftails, although two *Grallolichus* species are found in jacanas (Jacanidae; Charadriiformes). Finfoots have never been reported as hosts of *Grallolichus*. This study presents a description of a new species of the genus *Grallolichus* found for the first time on finfoots and, at the same time, it is the first new species of this genus described in the last 55 years.

## 2. Materials and Methods

The mite material was collected from an old museum specimen of the sungrebe *Heliornis fulica* (Gruiformes, Heliornithidae), preserved as an exhibit in the 19th-century ornithological collection of the Zoological Museum of Kiel University in Germany. Dried feather mites were carefully extracted by gently tapping the spread wing with a preparation needle holder over a piece of paper. The mites were stored in ATL buffer (Qiagen, Hilden, Germany) until they were subjected to DNA isolation according to the non-destructive method [35]. Mite exoskeletons were mounted on slides in Faure medium. Since all attempts at molecular analysis failed, the description of the species was made based on morphological characteristics only.

Morphological description of the new species follows recent standards for pterolichid species [36,37,38]. The nomenclature of idiosomal and leg chaetotaxy follows Gaud and Atyeo [4], but in nomenclature for coxal setation, we applied corrections proposed by Norton [39]. Drawings were made with a Leica DM5500 B microscope equipped with DIC illumination (Leica Microsystems Gmbh, Wetzlar, Germany) and a Olympus UD-A camera lucida (Olympus Corp., Tokyo, Japan). All measurements in the text and figures are in micrometres and are based on photos taken by a Leica DFC450 digital camera (Leica Microsystems GmbH, Wetzlar, Germany, 2015) and calculated by LAS v.4.6.1 software (Leica Microsystems GmbH, Wetzlar, Germany, 2015). Photos were made by LAS using Montage Multifocus option and modified in Helicon Focus 8.2.2. Pro software (Helicon Soft Ltd., Kharkiv, Ukraine, 2023). 

Measuring standards for structures are as follows:

(i) Idiosoma length is measured from the anterior margin of the propodosoma to posterior margin of opisthosomal lobes (males)/opisthosoma (females), including terminal membranes; width of idiosoma is the greatest width measured at the level of the sejugal region near humeral sclerites.

(ii) Hysterosoma length is measured from the level of lateral incisions of sejugal furrow to posterior margin of opisthosomal lobes (males)/opisthosoma (females), including terminal membranes. In males, hysteronotal shield length is measured from the level of anterior corners to bases of setae *h3*; in females it is separately measured for anterior main part and posterior pygidial part.

(iii) Distance between idiosomal setae of the same pair is the distance between their bases; distance between different pairs of setae is the distance between their bases of either side of the body (the mean of two measures for each specimen).

The bird classification and their scientific names are from Gill et al. [40].

## 3. Results

### 3.1. Systematics

Superfamily: Pterolichoidea Trouessart et Mégnin, 1884

Family: Pterolichidae Trouessart et Mégnin, 1884

Subfamily: Pterolichinae Trouessart et Mégnin, 1884

Genus: Grallolichus Gaud, 1960

### 3.2. Genus Diagnosis

Diagnosis is a compilation of the original genus definition of Gaud [18] and the corrected generic key of Pterolichinae prepared by Gaud and Atyeo [4]. The correction concerns the number of setae *vi*, because the original key does not include *Grallolichus jacanae* and *G. parrae* in their own genus.

Both sexes feature the following: legs without distinct apophyses, epimerites I fused; 0–2 filiform setae *vi*, setae *si* and *c2* filiform, not dilated; setae *c3* expanded, lanceolate and situated anteriorly to the long setae *cp*; solenidion *σ*1 on genu III present; setae *kT* on tibia IV present. Male: posterior legs not hypertrophied; opisthosoma bilobate; anal suckers with radiated corollas; genital papillae anterior to setae *4a*; genital organ located terminally, posterior to line joining articulations of trochanters IV. 

### 3.3. Species Description

**Figure 1:**
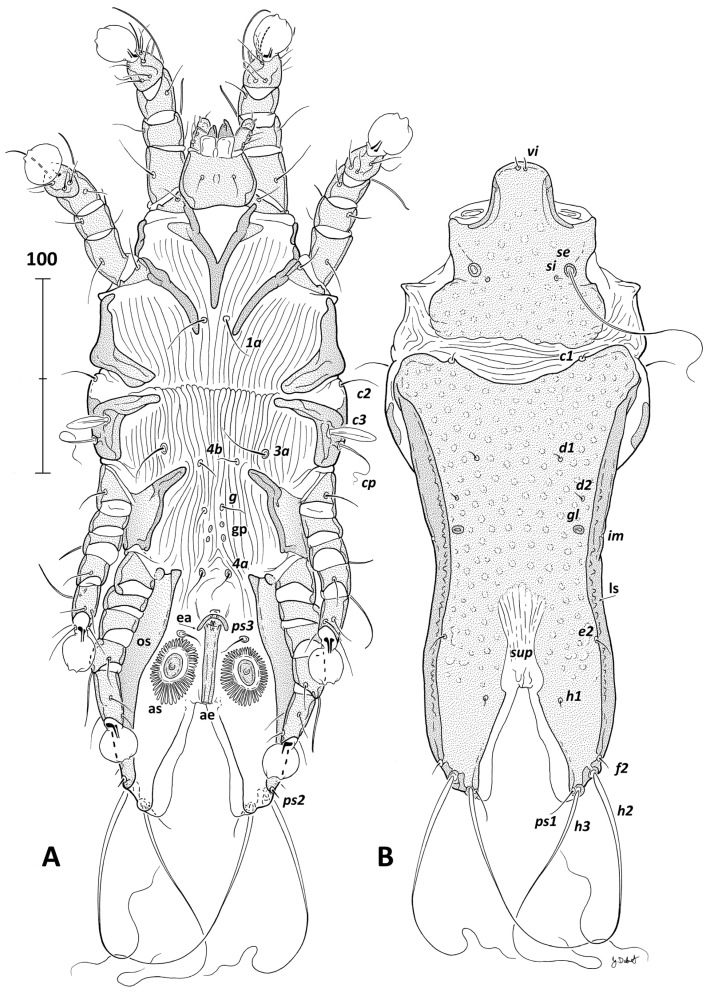
*Grallolichus heliornisi* sp. n., male. (**A**)—ventral view, (**B**)—dorsal view. Abbreviations: ae—aedeagus, as—anal suckers, ea—epiandrum, gp—genital papillae, ls—lateral sclerites, os—opisthoventral sclerites, sup—supranal concavity. Designation of setae and pores according to Gaud and Atyeo [4] with modifications by Norton [39].

**Figure 2:**
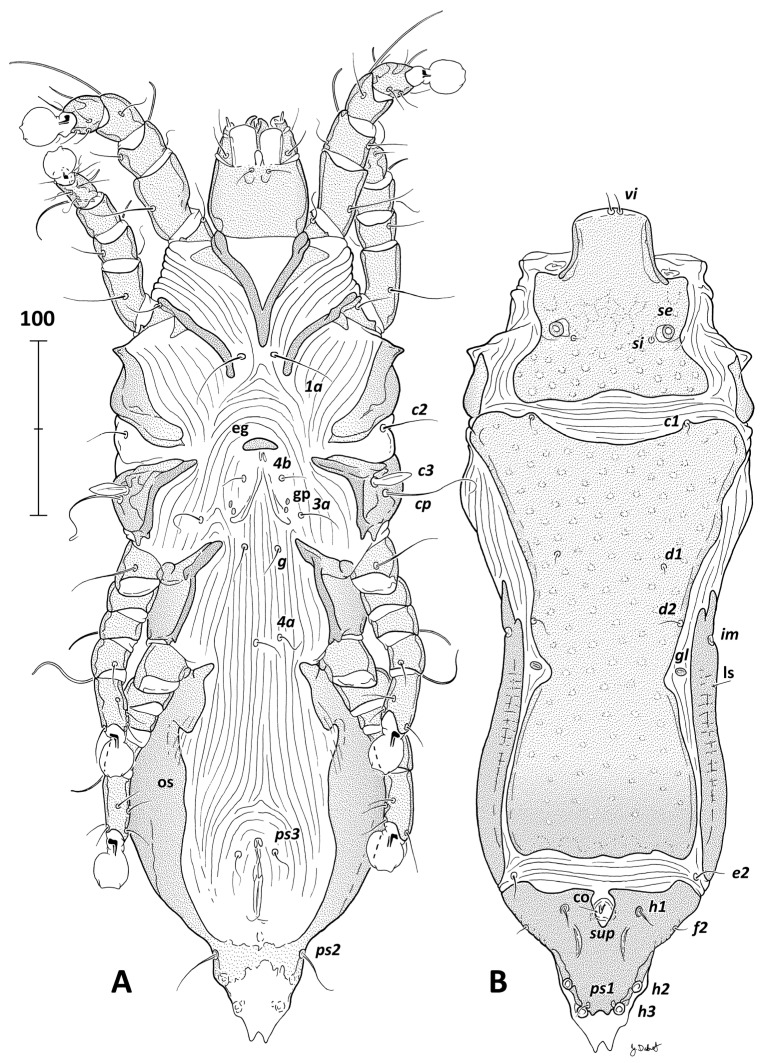
*Grallolichus heliornisi* sp. n., female. (**A**)—ventral view, (**B**)—dorsal view. Abbreviations: co—copulatory opening, eg—epigynum, gp—genital papillae, ls—lateral sclerites, sup—supranal concavity. Designation of setae and pores according to Gaud and Atyeo [4] with modifications by Norton [39].

**Figure 3:**
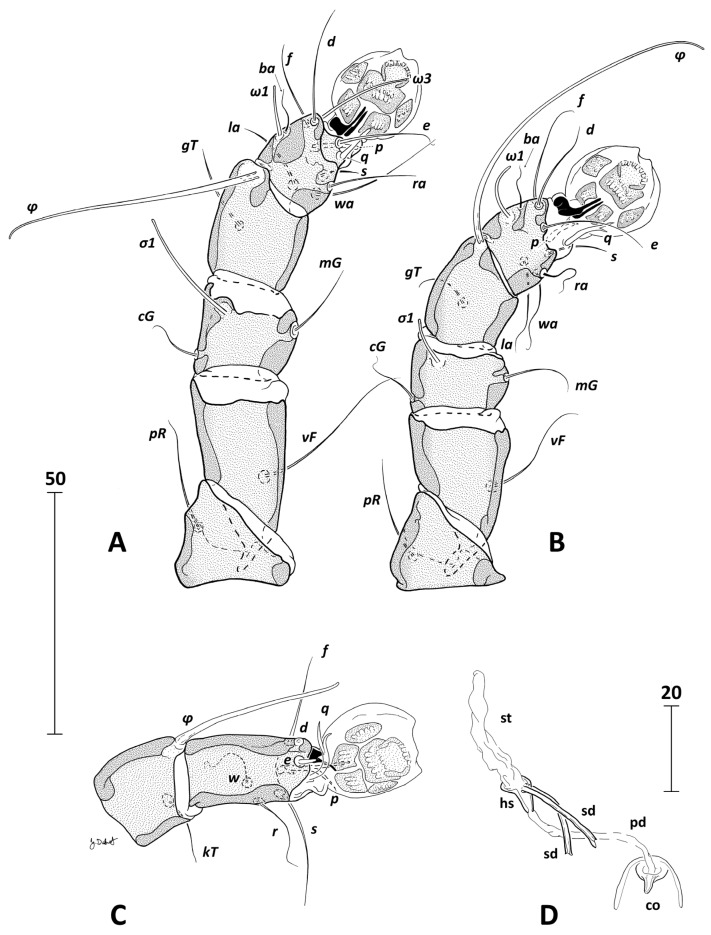
*Grallolichus heliornisi* sp. n., details. (**A**)—leg I, male, (**B**)—leg II, male, (**C**)—leg IV, male, (**D**)—spermatheca and spermaducts of female. Abbreviations: hs -head of spermatheca, pd—primary spermaduct, sd—secondary spermaduct, st—spermatheca. Setal designation according to Gaud and Atyeo [4].

***Grallolichus heliornisi* sp. n.** (Figure 1, Figure 2 and Figure 3).

**Type material**. Male holotype, three male, four female paratypes (AMU 01768) from the sungrebe *Heliornis fulica* (Boddaert, 1783), Brazil, October 1841, no other data, #A0796 (Zoological Museum CAU, Kiel, Germany), mites leg and det. J. Dabert.

**Description**. MALE (holotype, ranges for three paratypes in parentheses, Figure 1 and Figure 3A–C). Gnathosoma trapezoid, length including palps 49 (47–49), width at the base 42 (40–45). Body moderately elongated with well-developed opisthosomal lobes. Idiosoma, length × width 331 (312–328) × 140 (129–134); hysterosoma length 239 (219–238). Prodorsal shield trapezoid, with posterior margin irregularly waved and concavities near bases of setae *se*, surface with numerous rounded lacunes, length along midline 89 (86–91), width at posterior margin 93 (88–95) (Figure 1B). Internal vertical setae *vi* filiform, nearly as long as distance between them 3 (3–4), external vertical setae *ve* absent, represented by rudimentary alveoles. External scapular setae *se* long filiform, separated by 52 (50–55). Internal scapular setae *si* minute, not reaching corresponding bases of setae *se*, separated by 37 (36–39), set medial and slightly posterior to *se*. Scapular shields absent on dorsal propodosoma. Humeral shields narrow extending ventrally and fused to epimerites III. Setae *c2* filiform, set laterally out the anterior corners of hysteronotal shield, 20 (17–22). Setae *c3* lanceolate, 22 (17–21), set anteromedial to filiform setae *cp*, 37 (32–43). Hysteronotal shield shaped as inverted longitudinal trapezium, covers the whole hysteronotum, with concave anterior margin, length 232 (220–234), maximum width at anterior angles 120 (115–121); along whole lateral margins, darkly sclerotized bands (lateral sclerites) are present; surface ornamented as pronotal shield except posterior half of lobar region, which is uniformly dotted. Supranal concavity hourglass-like, large, open terminally and extending anteriorly in patch of desclerotized tegument with longitudinal striae. Triangular opisthosomal lobes well developed with slightly rounded lateral margins and straight median margins, length 55 (52–56), width at the base 46 (41–43). Interlobar cleft triangular, 55 (52–56) long, 48 (45–58) wide at the level of setae *ps1*. Narrow interlobar membranes present along the whole cleft, rounded terminally. All dorsal setae of hysteronotum filiform and very short, setae *e1* absent. Setae *c1* set on anterior margin of hysteronotal shield. Hysteronotal gland opening *gl* set posterior to setae *d2*. Fine cupules *im* present, set on lateral margins of hysteronotum slightly posterior to the level of *gl*. Setae *d1* on hysteronotal shield near its anterior margin. Setae *e1* absent. Setae *ps1* represented by microsetae. Distances between dorsal idiosomal setae and pores: *c1*:*d1* 53 (53–56), *d1*:*d1* 45 (39–49), *d2*:*d2* 67 (63–71), *d1*:*d2* 24 (21–22), *d2*:*e2* 74 (72–76), *e2*:*h1* 35 (36–39), *h1:h1* 42 (31–38), *d2*:*gl* 17 (15–19), *gl*:*e2* 58 (556–61), *h1*:*h2* 45 (41–50), *h2*:*h2* 72 (67–76), *h3*:*h3* 57 (50–64).

Epimerites I fused into a Y, sternum nearly half of the length of the whole epimerites (Figure 1A). All coxal fields open. Epimerites IVa with lateral shields connected with hysteronotal shield. Opisthoventral sclerites wide, extending from rudimentary epimerites IVa to the level of setae *ps2*. Genital region without sclerites. Genital organ inserted anterior to the level of setae *ps3*, three-times closer to these setae than to *4a*, and reaching posteriorly the anterior margin of the interlobar cleft. Aedeagus parallel-sided with the same diameter along the whole length, length 42 (40–42), width 6 (5–6) (Figure 1A). Epiandrum bow-shaped. Anal suckers elliptical with large, radiated corollas, corollas length 38 (34–39), width 25 (23–27), distance between centres of suckers 40 (37–41). Setae *4b* situated slightly posterior to level of setae *3a*. Genital papillae set equidistant to setae *g* and *4a* or slightly closer to *g* than to *4a*. Distances between ventral setae: *4b*:*3a* 15 (13–16), *4b*:*g* 26 (22–27), *g*:*4a* 34 (33–37), *4a*:*ps3* 34 (31–34); *ps*3:*ps2* 88 (86–87).

All legs of similar length and without apophyses (3A-C). Legs IV not reaching body terminus. All leg setae filiform except tarsal setae *p* and *q* and setae *d* and *e* on tarsi IV. Setae *p* and *g* on tarsi I are rod-like, on II-IV bifurcated. Setae *e* IV very short, stick-like, *d* IV reduced to flat button (Figure 3C). Solenidia *σ*1 I and σ1 III as long as genu I and genu III width, respectively, *σ*1 II twice shorter than genu II width. Solenidia *φ* I and II longer than tibia+tarsus I and II, respectively; *φ* III and IV shorter than tibia+tarsus III and IV, respectively.

FEMALE (range for four paratypes, Figure 2 and Figure 3D). Gnathosoma length 70–71, width 56–62. Body elongated, about 1.5 of male length, with distinctly protruded pygidial part provided with the terminal membrane. Membrane as long as distance *h3*:*h3*, split posteriorly into two small triangular teeth. Idiosoma length × width 480–484 × 170–184; hysterosoma length 364–368. Prodorsal shield shaped similarly as in male except desclerotized areas at medial margins of setae *se* (Figure 2B), posterior part with numerous rounded lacunes, median part with fine net-like sculpture, length 111–112, width 120–127. Vertical and scapular setae inserted as in males, *se* broken in all examined individuals, *si* minute as in males, *se*:*se* 65–73, *si*:*si* 45–53. Scapular shields as narrow longitudinal sclerites, humeral shields not visible on dorsal side of hysterosoma. Setae *c2*, *c3* and *cp* shaped as in males, 23–25, 22–25 and 57–61 long, respectively; *c2* set on ventro-lateral side of hysterosoma. Hysteronotal shield divided by transverse straight band of soft striated tegument situated on level of setae *e2* into main anterior part and posterior pygidial shield. Anterior part shaped and sculptured similarly to that in males but with distinct lateral incisions near gland opening *gl*, length 256–259, maximum width 161–171. Strongly sclerotized lateral sclerites separated from hysteronotal shield and extending from the level halfway between setae *d1* and *d2* to the level of setae *e2*. Pygidial shield strongly sclerotized with distinctly narrowed posterior part; length 73–77, maximum width 110–115. Supranal concavity open anteriorly and, therefore, anterior margin of pygidial shield interrupted. A pair of longitudinal folds is situated latero-terminally to posterior end of the supranal concavity. Copulatory opening set on a small triangular tubercle in the center of supranal concavity. Head of spermatheca funnel-like, 6–8 long, primary spermaduct of similar diameter along its entire length, relatively short (33–36); secondary spermaducts not enlarged distally, 23–23 long (Figure 3D). Dorsal setae of hysteronotum like those in males, setae *e1* absent, needle-like setae *h1* situated laterally to supranal concavity. Hysteronotal gland opening *gl* set posterior to setae *d2* on striated tegument. Well-developed cupules *im* present, set on lateral margins of hysteronotum posterior to the level of *d2* and anterior to *gl*. Distances between dorsal idiosomal setae and pores: *c1*:*d1* 80–83, *d1*:*d1* 64–76, *d1*:*d2* 38–43, *d2*:*d2* 88–98, *d2*:*e2* 144–145, *e2*:*h1* 36–38, *h1:h1* 43–43, *d2*:*gl* 28–32, *gl*:*e2* 112–117, *h1*:*h2* 42–47, *h2*:*h2* 40–45, *h3*:*h3* 20–25.

Epimerites I fused as in males, sternum about one-third of the length of whole epimerites (Figure 2A). Coxal sclerotization like that of male. Opisthoventral sclerites wide with incisions at level of genua IV, posteriorly connected to each other by weakly sclerotized band at the level of setae *ps2*. Epigynum as flat sclerite with rounded anterior margin and straight posterior margin, 6–6 high, 19–22 wide. Apodemes of oviporus without additional band-like sclerites. Setae *g* set at level of anterior ends of epimerites III, genital papillae slightly anterior to the level of *3a*. Distances between ventral setae: *4b*:*3a* 35–35, *4b*:*g* 42–47, *3a*:*g* 27–31, *g*:*4a* 46–49; *4a*:*ps3* 127–130, *ps*3:*ps2* 61–65, *ps*2:*ps2* 50–53.

Legs and solenidia shaped as in males, all setae filiform. Ambulacra of legs IV reaching the level of setae *ps3*.

**Depository**. Holotype and all paratypes are deposited in the Department of Animal Morphology, AMU, Poznań, Poland.

**Etymology**. The species epithet is taken from the generic name of the host and is a noun in the genitive case.

**Figure 4:**
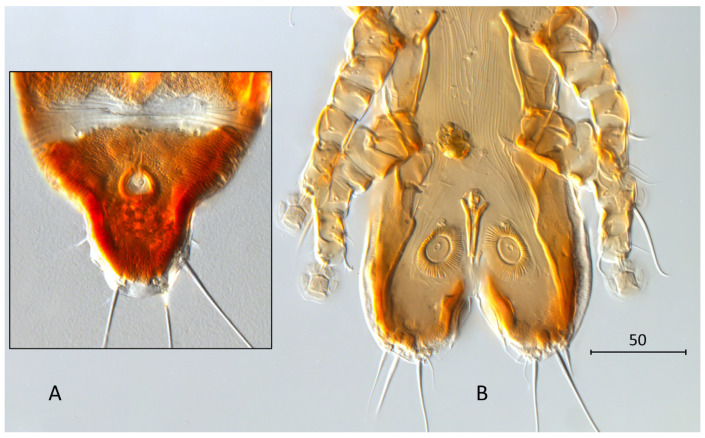
*Grallolichus proctogamus*. (**A**)—pygidial shield of female, (**B**)—genital region of male.

**Differential diagnosis.** The new species *Grallolichus heliornisi* sp. n. together with *G. cosmetonotus* Gaud, 1968 and *G. proctogamus* (Trouessart, 1885) belongs to the group of *Grallolichus* species having ornamented, ocellated dorsal shields in both sexes. The new species is most similar to *G. proctogamus* (Figure 4). Males of both species have a long aedeagus inserted near the level of setae *ps3*. Females have the posterior end of the body shaped like a tongue and elongated beyond insertion of setae *h2*, small supranal concavity and setae *h1* with sharp tips.

Males of the new species differ from *G. proctogamus* mainly by the shape of the opisthosomal region and form of the aedeagus. In *G. proctogamus*, opisthosomal lobes are as long as they are wide, slightly divergent posteriorly, and with widely rounded posterior margins. Opisthosomal lobes in *G. heliornisi* sp. n. are triangular and clearly longer than wide, parallel-sided with sharp posterior tips. Aedeagus in *G. proctogamus* is regularly tapering from the base to the distal end and does not reach the level of the posterior margin of anal suckers. In *G. heliornisi* sp. n., the aedeagus is almost parallel-sided along the whole length and reaches the level of the posterior margin of anal suckers. Females of *G. proctogamus* have closed, circular supranal concavity not connected with the anterior margin of the pygidial shield. In *G. heliornisi* sp.n., the supranal concavity is open anteriorly, making the anterior margin of pygidial shield interrupted. The terminal membrane of *G. proctogamus* is narrow without terminal incision, while in *G. heliornisi* sp. n., the membrane is well developed as a triangle with a small incision posteriorly. Setae *h1* in *G. proctogamus* are inserted on the soft tegument near the anterior margin of the pygidial shield, well anterior to the supranal concavity. In *G. heliornisi* sp.n., setae *h1* are inserted on the pygidial shield at the level of the supranal concavity.

### 3.4. Key for Grallolichus Species

This key is based on the one prepared by Gaud [14], with a modified setal nomenclature and some additions/corrections. Descriptions and illustrations (if any) of females of *G. dubinini*, *G. parrae*, and *G. amaurornis* are too brief to use in the key construction. The illustration of the *G. dubinini* male is detailed enough to apply in the key to males. The comparative material for *G. jacanae* and *G. proctogamus* originated from a synoptic collection from the Department of Animal Morphology, AMU.

**Males**

1. Setae *vi* absent ............................................................................................................*G. jacanae*– One or two setae *vi* present ....................................................................................................2.2. Single seta *vi*. Opisthosomal lobes long and pointed at their posterior end, separated by an interlobar cleft longer than double width of the lobe at its base ................. *G. aciurus*– Two setae *vi*. Lobes short, or with obtuse ends. Interlobar cleft shorter than double width of the lobe at its base ................................................................................................................. 3.3. Aedeagus base approximately at level of setae *4a* ...............................................................4.– Aedeagus base closer to setae *ps3* than to *4a* ........................................................................ 6.4. Aedeagus long (40 µm) and slender, with tip reaching behind the level of the adanal suckers ................................................................................................................. *G. melanurus*– Aedeagus shorter and thicker, tip not reaching setae *ps3* ................................................. 5.5. Dorsal shields ornate. Supranal concavity mace-like, open terminally and distinctly widened anteriorly ................................................................................................ *G. cosmetonotus*– Dorsal shields without ornamentation. Supranal concavity triangular, open terminally and narrowed anteriorly .............................................................................. *G. orthepigynius*6. Aedeagus shorter than half the diameter of the radiate corolla surrounding the adanal suckers ....................................................................................................................................... 7.– Aedeagus at least as long as than half the diameter of corolla ......................................... 9.7. Aedeagus triangular with posterior apex ........................................................................... 8.– Aedeagus rounded as wide as long ................................................................ *G. stagocaulus*8. Opisthosomal lobes well defined, at least 50 µm long ........................................ *G. benoiti*– Short opisthosomal lobes, less than 40 µm long ..................................................... *G. brevis*9. Aedeagus base anterior to the level of setae *ps3* .............................................................. 10.– Aedeagus base at the level or posterior to the level of setae *ps3* .................................... 11.10. Opisthosomal lobes slightly divergent with widely rounded posterior margins. Aedeagus regularly tapering from base to distal end .................................... *G. proctogamus*– Opisthosomal lobes triangular with sharp posterior tips. Aedeagus almost parallel-sided ...................................................................................................................... ***G. heliornisi* sp.n.**11. Aedeagus protruding well behind the adanal suckers into the interlobar cleft  *G. * *solenurus*– Aedeagus not extending posteriorly beyond level of adanal suckers ........................... 12.12. Aedeagus tips not reaching the level of anal suckers centers. Corolla of anal suckers elliptical, longer than wide .......................................................................... *G. eurytrematus*– Aedeagus tips at the level of anal suckers centers. Corolla of anal suckers circular .....13.13. Opistosomal lobes with parallel median margins in posterior half; lobes longer than their width at the bases ........................................................................................ *G. minutus*– Opistosomal lobes with triangular interlobar cleft; lobes shorter than their width at the bases ........................................................................................................................ *G. dubinini*

**Females**

1. Setae *vi* absent ............................................................................................................ *G. jacanae*– One or two setae *vi* present ................................................................................................... 2.2. Length less than 2.5 of width ............................................................................................... 3.– Length greater than 2.5 of width .......................................................................................... 4.3. Dark blade-like sclerites embedded deep in tissue from posterior end of oviporal apodemes ................................................................................................................... *G. brevis*– Absence of such sclerites .......................................................................................... *G. benoiti*4. Posterior end of body shaped tongue-like and elongated beyond insertion of setae *h2*, as long as or longer than the distance between *h2* ................................................................ 5.– No such body elongation. If the end of the body protrudes beyond the setae *h2*, it is much shorter than the distance between *h2* ........................................................................ 9.5. Very large supranal concavity, with transverse diameter equal to or greater than 25 µm ........................................................................................................................... *G. eurytrematus*– Transverse diameter of supranal concavity less than or equal to 10 µm ........................ 6.6. Setae *h1* dilated into small clubs ....................................................................... *G. melanurus*– Setae *h1* fine-tipped ................................................................................................................ 7.7. Dorsal shields ornate, covered by numerous rounded lacunes ...................................... 8.– Dorsal shields smooth with fine dots ................................................................................... 9.8. Supranal concavity open anteriorly and anterior margin of pygidial shield interrupted. Terminal membrane split, as long as distance between setae *h2*. Setae *h1* inserted on the pygidial shield at the level of supranal concavity ................................. ***G. heliornisi* sp.n.**– Supranal concavity closed and anterior margin of pygidial shield not interrupted anteriorly. Terminal membrane narrow without median incision. Setae *h1* inserted at the margin of pygidial shield anterior to supranal concavity ................................ *G. proctogamus*.9. Usually single seta *vi*. Transverse band of soft tegument between main part of hysteronotal shield and its pygidial part perpendicular to body axis and straight ........ *G. * *aciurus*– Usually two setae *vi*. Transverse band of soft tegument is shaped like a concave groove *G. stagocaulus*10. Setae *h1* inserted at the same level as the supranal concavity ................................ *G. * * solenurus*– Setae *h1* inserted anterior to the level of the supranal concavity ................................... 11.11. Hysteronotal shield relatively narrow, its posterior margin much shorter than the distance between setae *e2* .................................................................................. *G. orthepigynius*– Hysteronotal shield as wide posteriorly as distance between setae *e2* ......................... 12.12. Dark median longitudinal band, widening posteriorly, occupies the pygidial part of hysteronotal shield and bears the supranal cordiform concavity ............ *G. cosmetonotus*– Absence of a dark median band with distinct margins. Supranal concavity rounded *G.* * minutus*.

## 4. Discussion

It has long been noted that feather mites exhibit high host specificity at different taxonomic levels [41], sometimes serving even as additional information to identify their hosts. Also, many previous phylogenetic analyses have shown that numerous feather mite taxa show significant parallelism with the evolutionary history of their hosts, making them an important model for cophylogenetic studies [42,43], but there are also cases where little evolutionary parallelism is found when comparing the phylogenies of birds and feather mites [44,45]. However, such analyses, which are fundamental to understanding the evolutionary strategy of different groups of feather mites and their hosts, require good taxonomic sampling of both partners.

The degree to which the acarofauna of feather mites is known across bird taxa is very uneven. While most birds of Europe are well studied, non-European representatives of these taxa or birds specific to the remaining continents are much less studied or even completely unrecognized. According to rough calculations, the feather mite biodiversity of almost 30 bird families has never been studied, and for a further 50 families, mites have only been analyzed for 10% of bird species [46]. Estimating the global species diversity of feather mites is not straightforward. The available literature suggests that about 2500 of feather mite species can create more than 5000 mite–bird associations (JD, unpublished). Considering both monoxenic (single host) and polyxenic (multiple hosts) mite species and excluding erroneous literature data (accidental contamination, misidentifications), each bird species has, on average, two mite species of its own, resulting in 18,000–20,000 feather mite species. Assuming about 2500 feather mite species described [1], the recognition rate of this group of mites reaches 13–14%.

The *Grallolichus* species diversity is even less explored. With the species currently described, only 16 species of the genus are described for 176 potential ralloid and 8 jacanid host species [40]. Based on Table 1, *Grallolichus* species are probably more host-specific (ca. 1.3 mite species per host species) than average in all feather mites. Thus, so far, we know only about 7% of their potential species richness.

This number of described feather mite species would be even lower if the analysis of the feather mite acarofauna had to be based solely on examining living trapped birds or preserved specimens that died because of shooting or other lethal events, e.g., car accidents or crashing into obstacles, such as tall buildings, high-voltage power lines, or wind turbines. In the case of ectoparasites or commensals, which leave the host corpse after death, this is the only way to obtain material. However, feather mites and some other parasitic or commensalic mites (e.g., quill inhabiting Syringophilidae) remain in their host’s plumage as dried carcasses after the death of the host. Due to this behavior, a large proportion of the described feather mite species, including one presented here, derive from museum ornithological collections [47]. Ornithological collections are also the only source for obtaining and describing extinct feather mites from extinct hosts, the specimens of which are preserved in some exhibitions [48,49,50,51]. Old ornithological collections, sometimes in poor condition, are increasingly seen as a depleted source of ornithological research and hastily earmarked upon for decommissioning. However, they should still be preserved as an inexhaustible source for further research on the biodiversity and evolution of feather mites and other specific groups of ectoparasites or commensals.

## 5. Conclusions

This study presents the description of a new-to-knowledge species, *Grallolichus heliornisi* sp. n. (Pterolichidae), from sungrebe (Heliornithidae). This is the first description of a representative of this feather mite genus on the Heliornithidae, commonly found on other Ralloidea. At the same time, it is a description based on much more detailed morphological analyses than was standard 55 years ago, when the last of the 15 known species of the genus *Grallolichus* was described. These studies also gave rise to reflections on the importance of museum ornithological collections, which have been and continue to be an essential source of research into the biodiversity and evolution of feather mites.

## Figures and Tables

**Table 1:** Host associations and distribution of described *Grallolichus* species. The table is based on Doña et al. [13], with some necessary additions and corrections. Clear accidental associations or misidentifications found in the literature are not included. *—type host.

Mite Species	Host Species	Host Family	Locality	References
*G. aciurus* Gaud, 1968	*Sarothrura rufa* *	Sarothruridae	Dem. Rep. Congo, Cameroon	[14]
	*Sarothrura lugens lugens*	Sarothruridae	Dem. Rep. Congo	[14]
	*Sarothrura lugens lynesi*	Sarothruridae	Dem. Rep. Congo	[14]
*G. amaurornis* (Sugimoto, 1941) ^1^	*Amaurornis phoenicurus*	Rallidae	Taiwan	[15,16,17]
*G. benoiti* Gaud, 1960	*Himantornis haematopus*	Rallidae	Dem. Rep. Congo	[14,16,18]
*G. brevis* Gaud, 1960	*Himantornis haematopus*	Rallidae	Dem. Rep. Congo	[14,16,18]
*G. cosmetonotus* Gaud, 1968	*Rallus caerulescens*	Rallidae	Dem. Rep. Congo	[14]
*G. dubinini* (Vassilev, 1958)	*Gallinula chloropus*	Rallidae	Bulgaria	[19]
			Switzerland	[20,21]
			Poland	[22]
*G. eurytrematus* Gaud, 1968	*Canirallus oculeus*	Rallidae	Dem. Rep. Congo	[14]
*G. jacanae* (Gaud et Mouchet, 1959)	*Actophilornis africana*	Jacanidae	Cameroon	[16,23,24]
*G. melanurus* Gaud, 1968	*Sarothrura pulchra*	Sarothruridae	Dem. Rep. Congo	[14]
*G. minutus* Gaud et Mouchet, 1963	*Porphyrio alleni* *	Rallidae	Cameroon	[16]
			Dem. Rep. Congo	[14]
	*Porphyrio porphyrio*	Rallidae	South Africa	[16]
			Türkiye	[25]
	*Porphyrio madagascariensis*	Rallidae	Madagascar	[16]
			Rwanda, Dem. Rep. Congo,	[14]
			South Africa	
*G. orthepigynius* Gaud, 1968	*Aenigmatolimnas marginalis*	Rallidae	Dem. Rep. Congo	[14]
*G. parrae* (Mégnin & Trouessart, 1884)	*Hydrophasianus chirurgus*	Jacanidae	India	[16,26]
*G. proctogamus* (Trouessart, 1885)	*Fulica atra* *	Rallidae	France	[16,27]
			Russia	[28,29]
			Czech Republik	[30]
			Bulgaria	[19]
			Republic of Korea	[31]
	*F. cristata*	Rallidae	Rwanda, Dem. Rep. Congo	[16,24]
	*F. americana*	Rallidae	Canada	[32]
	*Gallinula chloropus* ^2^	Rallidae	Rwanda, Dem. Rep. Congo	[16,24]
	*P. madagascariensis* ^3^	Rallidae	Madagascar	[33]
*G. solenurus* Gaud et Mouchet, 1963	*Crex egregia*	Rallidae	Botswana Dem. Rep. Congo	[16] [14]
*G. stagocaulus* Gaud, 1968	*Sarothrura rufa* * Sarothrura lugens lugens *Sarothrura lugens lynesi*	Sarothruridae Sarothruridae Sarothruridae	Cameroon Dem. Rep. Congo Dem. Rep. Congo	[14] [14] [14]

^1^ Gaud and Mouchet [16] erroneously cited Sugimoto’s paper with the original description of the new species *Pterolichus amaurornis* when assigning it the genus *Grallolichus*. In fact, this was not the paper published in 1940 (Trans. Nat Hist. Soc. Formosa), but in 1941 (Sylvia). ^2^ Gaud [14] stated that the specimens of *G. proctogamus* he found on *Gallinula chloropus* are very different from the mites from *Fulica*. In the former, the pygidial shield of females is less trilobed and more triangular. In addition, the ornamentation of the dorsal shields is different: the groups of spots present in *G. proctogamus* are replaced by small dots. It is likely that Gaud was dealing with *G. dubinini*, but this question could be resolved only after the revision of this species, as the original description prepared by Vassilev [19] is too cursory and poorly illustrated. ^3^ Gaud [33] reported the species *Pterolichus digamus* from this host in Madagascar, without description or illustration. Doña and collaborators [13] considered it to be *Grallolichus proctogramus*. However, Dubinin [29] and Mironov [34] showed that *P. digamus* is a group of two species originally described from *Fulica atra*: *Grallobia fulicae* and *Grallolichus proctogamus*. It is therefore not possible to determine which of these species, if any, Gaud was dealing with. It is quite possible that it could have been the later-described *G. minutus* [16].

## Data Availability

All necessary details of the material described, including locations, dates and the name of the collector, are available in this article. Upon reasonable request, the material can be made available by the author.
